# A Hard Case

**Published:** 1888-04-28

**Authors:** Margaret Niven

**Affiliations:** Battersea, S.W.


### The Editor's Letter-Box.

[Our Correspondents are reminded that prolixity is a great bar to publication, and that brevity of style and conciseness of statement greatly facilitate early insertion.]

### A HARD CASE.

I am exceedingly troubled just now about a very difficult case of paralysis, which no hospital will take in even as a paying patient. The patient's income will not permit of the expense of a nurse at the ordinary rates for such cases—indeed her means are so limited she cannot afford a nurse at any figure; but her landlady can hardly continue the strain of attending on her much longer without trained help. The patient is in the South of England, and could not be removed. Is there any nursing sisterhood who take up such cases as their special care? Can you in any way help me, through your paper or otherwise? I shall be grateful if you can. The patient is an educated lady, daughter of a clergyman.

Battersea, S.W. Margaret Niven.

